# Quantification of facial cues for acute illness: a systematic scoping review

**DOI:** 10.1186/s40635-025-00719-x

**Published:** 2025-02-13

**Authors:** Iris C. Cramer, Eline G. M. Cox, Jip W. T. M. de Kok, Jacqueline Koeze, Martje Visser, Hjalmar R. Bouma, Ashley De Bie Dekker, Iwan C. C. van der Horst, R. Arthur Bouwman, Bas C. T. van Bussel

**Affiliations:** 1 Department of Electrical Engineering, Eindhoven University of Technology, Eindhoven, The Netherlands; 2 Department of Anesthesiology and Pain Medicine, Catharina Hospital, Eindhoven, The Netherlands; 3 Department of Intensive Care Medicine, Maastricht University Medical Centre+, Maastricht, The Netherlands; 4 Care and Public Health Research Institute CAPHRI, Maastricht University, Maastricht, The Netherlands; 5 Cardiovascular Research Institute Maastricht CARIM, Maastricht University, Maastricht, the Netherlands; 6 Department of Critical Care, University Medical Center Groningen, Groningen, The Netherlands; 7 Department of Internal Medicine, University Medical Center Groningen, Groningen, The Netherlands; 8 Department of Acute Care, University Medical Center Groningen, Groningen, The Netherlands; 9 Department of Clinical Pharmacy & Pharmacology, University Medical Center Groningen, Groningen, The Netherlands; 10 Department of Intensive Care, Catharina Hospital, Eindhoven, the Netherlands; 11 Department of Intensive Care, Anna Hospital, Geldrop, the Netherlands

**Keywords:** Facial cues, Clinical gestalt, Facial appearance, Deterioration detection, Monitoring

## Abstract

**Importance:**

The patient’s face provides healthcare professionals with important information about the patient’s general appearance and clinical condition.

**Objective:**

The primary aim of this review is to identify patients’ facial cues that healthcare providers can use at the bedside to monitor the clinical condition of acutely ill patients.

**Evidence review:**

Studies about facial cues for acute illness were systematically searched in PubMed, Embase, Cochrane, and Cumulative Index to Nursing & Allied Health (CINAHL) databases. Studies on vital signs, pain, psychiatric illnesses, animal studies, qualitative studies, case reports, and systematic reviews were excluded. Acute illness was defined as any life-threatening condition or condition that required immediate intervention to prevent serious morbidity, permanent disability, or mortality. An overview of all identified facial cues was created.

**Findings:**

In total, 35 different facial cues were identified in 13 studies. A total of 21 were related to facial appearance, with the most frequently reported cues being closed eyes (2 studies), pale lips (2 studies), parted lips (3 studies), droopy mouth (3 studies), and paler skin tone (2 studies). In addition, 14 facial expression features were identified, characterized primarily by more sad, less happy, and less surprised. Most cues have only been described in a single study without external validation, limiting the generalizability of definitions of these cues and their clinical applicability.

**Conclusions and relevance:**

This systematic scoping review identified 35 facial cues associated with acute illness in patients in the hospital, highlighting the potential of facial observation to enhance clinical assessments. However, the lack of standardization limits applicability in healthcare. Future research should refine the setting of acute illness, develop diverse datasets, and validate the predictive value of facial cues across various populations.

**Supplementary Information:**

The online version contains supplementary material available at 10.1186/s40635-025-00719-x.

## Introduction

Every encounter between healthcare providers and patients offers a unique chance to collect data about the patient’s general appearance [1]. The patient’s appearance plays a role, often subconsciously informing the healthcare providers whether the patient seems unwell to estimate the severity of their illnesses [2]. The ability to recognize, interpret and objectively monitor facial cues in acutely ill patients provides valuable clinical information regarding the course of their illness and may aid in the rapid assessment and management of their condition [3]. Studies have demonstrated that healthcare providers can effectively use facial appearance to assess pain, distress, and overall clinical status in acutely ill patients [3–5]. Moreover, incorporating facial cues into machine-learning models may enhance the assessment of a patient’s health status [6].

Before integrating facial features into regular patient monitoring, it is crucial to define the facial cues and determine which cues are useful in a specific clinical setting. Currently, there is no comprehensive overview of the predictive value of facial cues for acute illness in the acute care setting. Although various studies have explored signs and symptoms that reflect clinical gestalt—the healthcare professional’s judgment regarding a patient's risk of deterioration—most of these investigations have been predominantly qualitative. This limitation hampers the generalization of findings and the quantification of facial cues' predictive value [7]. Although numerous reviews have demonstrated the prognostic value of vital parameters, a comprehensive synthesis of the predictive potential of specific facial cues for patient outcomes remains absent. Therefore, the primary aim of this systematic scoping review was to identify the facial cues at the bedside that suggest an acute illness of patients in the hospital.

## Methods

### Protocol and registration

We have reported all applicable PRISMA-ScR items (Supplementary Material 1) [8]. Notably, we aimed to publish the protocol in PROSPERO on June 26, 2024, but during the process, it showed that PROSPERO currently does not accept registrations for systematic scoping reviews, literature reviews, or mapping reviews.

### Search strategy

PubMed, Embase, Cochrane, and Cumulative Index to Nursing & Allied Health (CINAHL) databases were searched for relevant articles published until 27–03-2024, according to the PRISMA statement methodology [9]. The full search strategy is presented in Supplementary Material 2. In addition, we performed a snowballing approach to ensure comprehensive coverage of all relevant literature. This method allows us to identify important studies that may not have appeared in the initial database search but are cited by or referenced by other key studies. Snowballing was conducted to minimize the risk of missing relevant studies, ensuring our systematic review's robustness and completeness.

Duplicate articles were identified by Rayyan—a web and mobile app for systematic reviews (http://rayyan.qcri.org)—and removed [10]. Two reviewers (IC and EC) independently screened each title and abstract and included those deemed relevant. The full texts of the remaining studies were retrieved, read, and assessed based on the eligibility criteria. Disagreements were resolved through discussion between the two reviewers (IC and EC), and a third assessor was consulted if consensus could not be reached (BvB).

### Eligibility criteria

The eligibility criteria are listed in Supplementary Material 3. Briefly, we included studies of any language concerning adult patients in an in-hospital setting that evaluated acute illness based on facial cues. Acute illness was defined as any life-threatening condition or condition that required immediate intervention to prevent serious morbidity, permanent disability, or mortality. Studies on psychiatric illnesses, animal studies, qualitative studies, case reports, and systematic reviews were excluded. Next, studies evaluating only vital signs were excluded as this is not the topic of the present investigation, and there is an existing body of evidence on vital signs for critical illness and monitoring [11–14]. Importantly, vital signs are not visible signs but measurements. In addition, studies that used pain as an outcome measure were excluded.

### Data extraction

The data were extracted by two reviewers (IC and EC). The extracted data included study characteristics, study population, outcomes, methodology, and facial cues. Supplementary Material 4 lists the prespecified data items to be extracted. An overview of all identified facial cues was created using Microsoft Excel version 16.75, Microsoft Corporation, Redmond, Washington, USA.

## Results

### Study selection

**Fig. 1:**
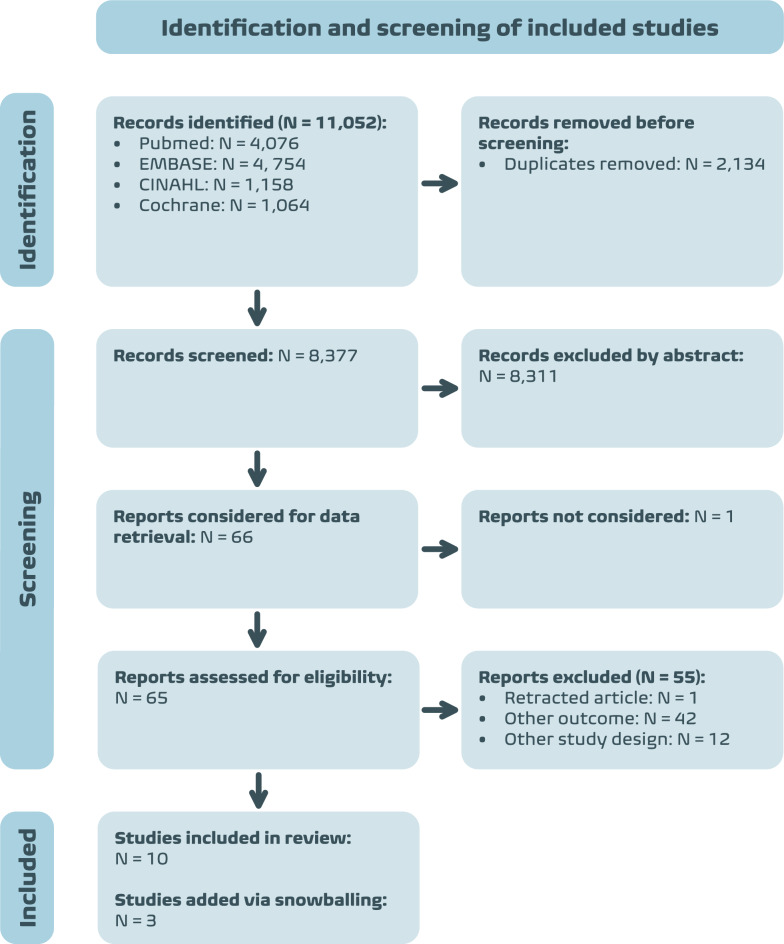
PRISMA flowchart showing the identification and screening of the included studies. PRISMA: Preferred Reporting Items for Systematic reviews and Meta-Analyses

From the systematic literature search of PubMed, Embase, CINAHL, and Cochrane Central Register of Controlled Trials (CENTRAL) databases, we identified 8377 studies that were screened based on the title and abstract (Fig. 1). As a result, 66 studies were evaluated for their full text. Most studies (85%) were excluded based on their outcomes. These studies predominantly did not quantify or specify the criteria upon which clinical gestalt was based. Ultimately, 10 studies were included in this review. Additionally, by snowballing (i.e., using the reference lists of the 10 included studies), three other studies were added.

### Characteristics of the included studies

Studies were published in the United States (31%), the Netherlands (31%), the United Kingdom (23%) and Sweden (15%) (Table 1). In four studies (31%), healthcare providers evaluated facial cues. Five studies (38%) analyzed photographs, and four studies (31%) investigated facial cues in video monitoring. Only one study included a mixed ethnicity, while the other studies either did not address this topic or included only Caucasian participants.

**Table 1:** Characteristics of the included studies according to year of publication

Author, year	Country	Identification method	Study design	N	Method to obtain visual cues*		
					Photographs	Video	Healthcare providers
Dalton, 1999 [15]	US	Snowballing	Observational prospective	28	X		
Rosenberg, 2001 [16]	US	Search	Video-taped structured interview	115		X	
Kline, 2015 [17]	US	Snowballing	Observational prospective	50		X	
Douw, 2016 [18]	Netherlands	Snowballing	Exploratory study	3522			X
Henderson, 2017 [19]	UK	Snowballing	Randomized experiment	22	X		
Axelsson, 2018 [5]	Sweden	Search	Randomized experiment	22	X		
Margridal-Garcia, 2018 [20]	UK	Search	Observational prospective	34		X	
Sarolidou, 2019 [21]	Sweden	Search	Randomized experiment	22	X		
Madrigal-Garcia, 2020 [22]	UK	Search	Observational prospective	43		X	
Castela Forte, 2021 [6]	Netherlands	Search	Validation study with a machine learning approach	26	X		
Shiber, 2023 [4]	US	Search	Observational prospective	216			X
Visser, 2024 [23]	Netherlands	Search	Observational prospective	517			X
Cox, 2024 [3]	Netherlands	Snowballing	Observational prospective	228			X

US: the United States; UK: the United Kingdom

^*^The category was divided into three subcategories based on how the cues were measured: photographs, videos, and healthcare providers

### Outcome measures

The definition of clinical outcome varied among the studies (Table 2). Acute illness was defined by various criteria, such as ischemia, mortality < 72 h, and unplanned ICU admission. Four studies (31%) reported a combination of adverse events as outcomes. Four studies (31%) reported that lipopolysaccharide (LPS)-induced illness in healthy volunteers was the outcome. Two studies (15%) reported cardiac ischemia as an outcome. The included studies show that various facial cues were able to identify deterioration of the patient’s clinical condition. For example, facial cues such as decreased redness, paler lips, and tired appearance were related with acute illness. Also, patterned facial expressions, including increased sadness and disgust, and decreased happiness and surprise, were observed in deteriorating ward patients. The methodologies used to quantify the accuracy of detecting acute illness varied widely, which made pooling results across studies not feasible.

**Table 2:** Aim, outcome definitions, and results of the included studies

Author, year	Aim	Outcome definition	Results
Dalton, 1999 [15]	1) To identify the facial expressions of adult patients complaining of chest pain who are admitted to the emergency department with a possible diagnosis of acute ischemic heart disease (AIHD) 2) To discover what facial expressions are exhibited by patients complaining of chest pain with a possible diagnosis of AIHD and whether those cues are related to other commonly used measures or predictors of AIHD that can aid in the diagnosis of myocardial infarctions	Creatine kinase enzyme-positive myocardial infarction	Facial expressions were found to be associated with true myocardial infarction: lowering the brow, pressing the lips, parting the lips, and turning the head left
Rosenberg, 2001 [16]	To assess if dynamic indicators of emotional cues related to coronary changes (ischemia)	Cardiac ischemia is reflected as a disturbance in wall motion abnormality and/or changes in left ventricular ejection fraction obtained by radionuclide ventriculography	Participants with ischemia showed more anger expression and nonenjoyment smiles than non ischemics
Kline, 2015 [17]	To determine if patients who are more acutely ill have less facial expression variability in response to emotional cues	Serious cardiopulmonary diseases on computed tomography angiography	With a single visual stimulus, patients with serious cardiopulmonary diseases lacked facial expression variability and surprise affect
Douw, 2016 [18]	To quantify nurses’ ‘worry’ and indicators underlying ‘worry’ (DENWIS indicators) to predict unplanned ICU admission or unexpected mortality among surgical ward patients	Unplanned ICU admission or unexpected in-hospital mortality	DENWIS indicators improved prediction of unplanned ICU admission or mortality
Henderson, 2017 [19]	To determine possible skin colour changes to detect illness	LPS-induced illness	The face lost redness and became lighter with acute sickness
Axelsson, 2018 [5]	To determine whether it is possible to identify experimentally induced ill people based on facial pictures and to specify cues that contribute to this identification	LPS-induced illness	Acutely sick people were rated by naive observers as having paler lips and skin, a more swollen face, droopier corners of the mouth, more hanging eyelids, redder eyes, and less glossy and patchy skin, as well as appearing more tired
Margridal-Garcia, 2018 [20]	To identify facial expressions in patients at risk of deterioration in hospital wards	ICU admission	Patterned facial expressions can be identified in deteriorating general ward patients
Sarolidou, 2019 [21]	To test whether volunteers who underwent experimentally-induced illness expressed different facial emotions, as judged by naïve raters, compared to the same volunteers in the control condition	LPS-induced illness	Sick faces are perceived as more disgusted and sad, and less happy and surprised
Madrigal-Garcia, 2020 [22]	To determine whether the amount and diversity of facial expressions predict acute clinical deterioration in patients in general hospital wards	ICU admission, in-hospital mortality	Patients who will be admitted to intensive care have a decrease in the number of facial expressions per unit of time and an increase in their diversity
Castela Forte, 2021 [6]	To assess whether a deep learning algorithm trained on a dataset of simulated and augmented facial photographs reflecting acutely ill patients can distinguish between healthy and LPS-infused, acutely ill individuals	LPS-induced illness	A deep learning algorithm can distinguish between healthy and simulated sick individuals (AUC 0.67)
Shiber, 2023 [4]	To investigate whether a clinical judgment accurately predicts the severity of injury or illness and whether it can be used at patient arrival when other formal scoring systems are not yet available	Immediate disposition from the emergency department or trauma centre: Home, Brief Observation (< 24 h), Admission to the ward, ICU, or Morgue	Physicians can make accurate predictions of severity of illness using the GCCS score
Visser, 2024 [23]	To investigate the subjective features and objective measurements that determine the clinical impression of the health care professional at the emergency department	Primary: Clinical impression score, scale 1–10: how ill is the patient? Secondary: Admission to hospital, ICU admission, in-hospital mortality (< 48 h), and 28-day mortality	Dry mucous membranes, eye glance, red flags during physical examination, results of arterial blood gas analysis, heart and respiratory rate, oxygen modality, triage urgency, and increased age were associated with a higher estimated disease severity
Cox, 2024 [3]	To investigate whether facial appearance at admission is associated with longitudinal evaluation of multi-organ failure	Daily longitudinal SOFA score	Facial appearance scored by the extent of eye-opening was associated with a higher SOFA score at admission and follow-up. There was no association between facial skin colour and a worse SOFA score over time. However, patients with half-open or closed eyes along with flushed skin had a lower SOFA score than patients with a pale or normal facial skin colour

LVEF: Left ventricular ejection fraction; ICU: Intensive Care Unit; SQ-score: Self-reported illness score; LPS, liposaccharide; SOFA: Sequential Organ Failure Assessment, DENWIS: Dutch Early Nurse Worry Indicator Score, GSSC: Gestalt Clinical Severity Score

### Facial cues

**Fig. 2:**
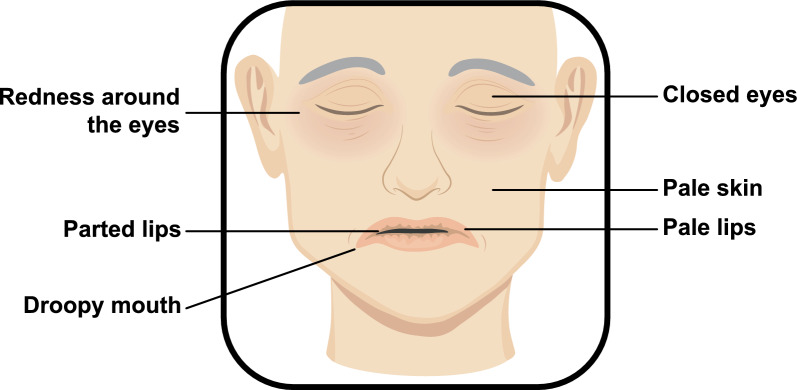
Most frequently reported facial cues for acute illness

**Fig. 3:**
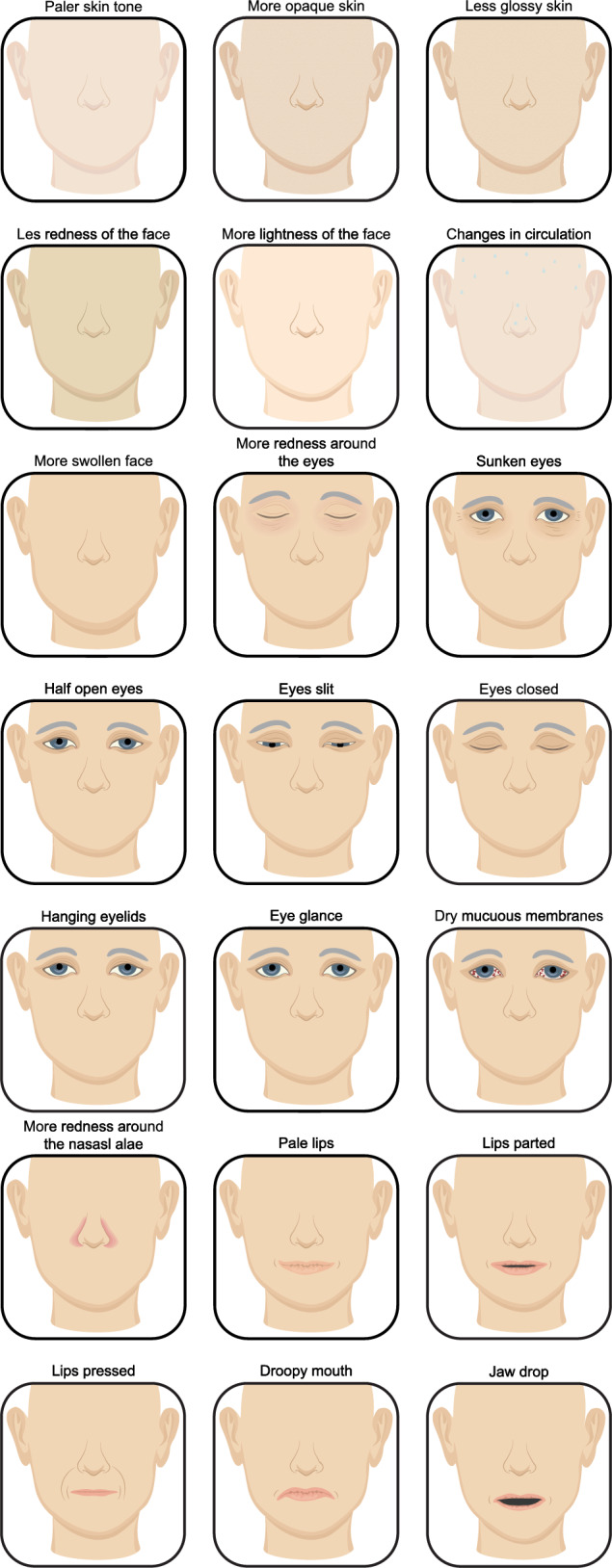
Figure of all facial cues for acute illness

The identified facial cues were categorized into two main groups: (1) facial appearance; and (2) facial expressions (Table 3, Fig. 2,3). The distinction was made because facial appearance refers to more static, structural cues, often involving a single changed cue, while facial expressions focusses on dynamic, emotional responses, like sadness or fatigue, which typically involves a combination of changes cues. This separation enables a clearer analysis of both structural and emotional cues when recognizing acute illness.

**Table 3:** Facial cues for acute illness

Facial cues for acute illness	Dalton, 1999 [15]		Rosenberg, 2001 [16]		Kline, 2014 [17]		Douw, 2016 [18]		Hendersson, 2017 [19]		Axelsson, 2018 [5]		Margridal-Garcia, 2018 [20]		Sarolidou, 2019 [21]		Madrigal-Garcia, 2020 [22]		Castela Forte, 2021 [6]		Shiber, 2023 [4]		Visser, 2024 [23]		Cox, 2024 [3]
Facial appearance																									
Skin																									
Paler skin tone										x								x							
More opaque skin																		x							
Less glossy skin										x															
Less redness of the face								x																	
More lightness of the face								x																	
Changes in circulation						x																			
More swollen face										x															
Eyes																									
More redness around the eyes										x								x							
Sunken eyes																		x							
Half-open eyes																								x	
Eyes slit																								x	
Eyes closed												x^a,b,c^												x	
More hanging eyelids										x															
Eye glance																						x			
Dry mucous membranes																						x			
Mouth and nose																									
Pale lips										x								x							
Lips parted	x											x^a^						x^a^							
Lips pressed	x																								
Droopy mouth										x		x^a,b,c^						x							
Jaw drop	x																								
More redness around the nasal alae																		x							
Skin																									
Paler skin tone										x								x							
More opaque skin																		x							
Less glossy skin										x															
More swollen face										x															
Less redness of the face								x																	
More lightness of the face								x																	
Changes in circulation						x																			
Facial expressions																									
More sad										x				x											
More disgusted														x											
Less happy														x											
More surprised														x											
Less facial variability: surprised				x																					
Less facial variability: frowning				x																					
Less facial expression																x									
More tired										x															
Non-enjoyment smiles		x																							
More angry		x																							
Head turned left or right	x											x^a,b,c^				x^a,b,c^									
Eyes closed, mouth hanging open																				x					
Eyes open, grimace on the face, appears distressed																				x					
Eyes open + flat affect + unequivocal facial expression																				x					

Combination of: ^a^ Eye closure + lip corner depression + lips parted

^b^Eye closure + lip corner depression + head turned left or right

^c^Eye closure + lip corner depression + head turned left

#### Facial appearance

Acutely ill patients were found to have dry mucous membranes, increasingly red eyes, and lower eye glance [5, 23]. Moreover, they closed their eyes and had hanging eyelids more often [3, 5]. Naive observers rated acutely ill patients as having paler lips and skin, a more swollen face, and droopier corners of their mouths (Figs. 2,3) [5, 20]. Also, acutely ill patients exhibit increased redness around the nasal alae. One study has indicated that visible changes in circulation, such as colour changes, skin clamminess, coldness, impaired perfusion, and oedema, suggest acute illness [12].

#### Facial expressions

Patterns of facial expressions can be identified in deteriorating general ward patients [20]. Facial expressions of acutely ill patients are perceived as more sad, less happy, and less surprised [21]. Patients admitted to the ICU have a decrease in the overall number of facial expressions and an increase in the diversity of facial expressions [22]. In addition, acutely ill patients appear more tired [5].

## Discussion

This systematic scoping review identified 35 distinct facial cues associated with acute illness of patients in the hospital. Of these, 21 cues related to facial appearance, with the most frequently reported cues being closed eyes, pale lips, parted lips, droopy mouth, and paler skin tone. In addition, 14 facial expression features were identified, characterized by expressions of sadness, less happiness and surprised. The outcome definitions varied across studies, and facial cues were extracted using various methods, including photography, video monitoring, and healthcare provider assessments.

While this review highlights the promise of facial cues in clinical assessments, their current ability to reliably detect changes in clinical condition remains uncertain. Results from qualitative studies support the promise of these cues, aligning with our results [7]. However, existing evidence mostly includes facial cues at a single time point, ignoring dynamic trends. This is an important limitation, as clinical deterioration often occurs gradually with subtle changes over time. For example, facial cues, such as a paler skin, droopier mouth corners, or reduced facial expression variability have been associated with acute illness, although it remains unclear whether these features can reliably capture progressive changes over time. Moreover, the balance between sensitivity and specificity is a challenge. Systems that are overly sensitive may result in frequently false positives, overwhelming healthcare providers with unnecessary alarms. Conversely, systems with low sensitivity, designed to detect extreme cases only, risk missing initial, more subtle signs of deterioration, delaying critical interventions.

## Strengths and limitations

While this review provides valuable insights into the facial cues associated with acute illness, several limitations should be acknowledged. First, the heterogeneous nature of the definition of “acute illness” as an outcome complicates the pooling and comparison of the results. Furthermore, it remains unclear whether this term is synonymous with other concepts such as clinical impression, gut feeling, or gestalt. Variability in the descriptions of facial cues and the use of diverse study designs further limit the generalizability of findings. Additionally, capturing all relevant facial cues through a comprehensive search is inherently challenging, possibly leading to an incomplete representation of the available evidence. However, by employing the snowballing technique—whereby references from included studies were reviewed to identify additional relevant research—we believe we have mitigated this limitation as much as possible. Importantly, we feel that potentially missed studies will not change the overall conclusion that facial cue monitoring is promising and should be investigated further. Another limitation is the geographic focus of the studies included. All were conducted in Western countries. This may restrict the generalizability of the findings, as facial cues might present differently across diverse populations with varying skin tones and facial characteristics. Understanding how these cues manifest in people with different ethnic backgrounds is essential for the broader application of future facial cue monitoring.

## Future research and implications for clinical practice

To implement these findings in clinical practice, future research should begin by defining the setting with acute illness. The predictive value of the identified facial cues needs further evaluation, and facial cues should be deconstructed into individual components that can be objectively quantified, possibly using machine learning models. The diversity of cues presents a challenge in determining their individual and collective value in predicting patient deterioration. Moreover, the identified facial cues can serve as a starting point for the development of novel camera monitoring systems capable of detecting these cues continuously and in real-time. Incorporating facial cues into clinical decision support systems might enhance early detection and offer additional value by leveraging the clinical gestalt—“the ability of a clinician to integrate combinations of clinical signs, symptoms, and other information to arrive at an aggregate appraisal”. Additionally, artificial intelligence may be able to detect patterns in the face that are not perceptible to humans, which could be particularly valuable for identifying clinical deterioration. These capabilities could complement current clinical decision support systems, which predominantly rely on physiological parameters and vital signs but ofen suffer from low predictive accuracy, leading to issues such as alarm fatigue [24, 25]. This approach will require extensive datasets with clearly defined endpoints to train and validate artificial intelligence models. Future studies should also integrate facial cues into clinical decision support systems to evaluate their utility in real-time patient monitoring and early detection of deterioration. Importantly, diverse populations must be included to ensure that the identified facial cues are universally applicable and that clinical decision support systems can be adapted to recognize and interpret these cues accurately in all patient groups.

## Conclusions

This systematic scoping review identified 35 facial cues linked to acute illness, highlighting their potential to enhance clinical assessments. However, the lack of standardization and external validation limits their current applicability. Future research should refine the definition of acute illness, validate facial cues across diverse populations, and explore the integration of AI-driven technologies for real-time monitoring.

## Supplementary Information

Additional file 1.

## Data Availability

The datasets used and/or analyzed during the current study are available from the corresponding author upon reasonable request.
